# A Novel MR Elastography Device for Preclinical Applications From Spheroids to Rodents: Design, Validation, and Performance Evaluation

**DOI:** 10.1002/nbm.70416

**Published:** 2026-10-06

**Authors:** Gabrielle Mangin, Hannah Fels‐Palesandro, Valéria Bisio, Nicolas Dulphy, Karl Balabanian, Inas H. Faris, Andrea Federici, Asma Boumaza, Marguerite Ducamp, Roland Zerelles, Valérie Vilgrain, Valérie Paradis, Giacomo Annio, Katharina Schregel, Ralph Sinkus

**Affiliations:** ^1^ Center for Research on Inflammation, INSERM UMR 1149 University Paris Cité Paris France; ^2^ Assistance Publique—Hôpitaux de Paris Beaujon Hospital, AP‐HP Clichy France; ^3^ Department of Neuroradiology Heidelberg University Hospital Heidelberg Germany; ^4^ Institut de Recherche Saint Louis, INSERM UMR1342 Université Paris Cité Paris France; ^5^ Laboratoire d'Immunologie et Histocompatibilité, Assistance Publique—Hôpitaux de Paris Saint‐Louis Hospital, AP‐HP Paris France; ^6^ Department of Structural Mechanics University of Granada Granada Spain; ^7^ School of Biomedical Engineering and Imaging Sciences King's College London London UK; ^8^ Institute for Radiology, Neuroradiology and Nuclear Medicine Klinikum Darmstadt Darmstadt Germany; ^9^ Department of Radiology, Section of Neuroradiology Jena University Hospital Jena Germany

**Keywords:** elastography, MRE, preclinical MRE applications, tissue biomechanics

## Abstract

Magnetic resonance elastography (MRE) is a powerful technique for assessing tissue biomechanics noninvasively. To disentangle and understand the histopathological and cellular signature of changes in tissue biomechanics, preclinical studies remain indispensable. Yet, the preclinical research setting for MRE is currently characterized by a multitude of different hardware solutions designed by different research groups that are often tailored to a single preclinical application. This impedes comparability and reproducibility between the results obtained within the community. This study presents the design, fabrication, and validation of a novel multipurpose preclinical MRE device allowing the examination of rodents, ex vivo tissue samples, and spheroids/organoids. Validation, stability, and reproducibility experiments were conducted on ultrasound gel phantoms. Highly reproducible maps of tissue mechanics are also demonstrated in rodent models and ex vivo biological samples, showing a high degree of concordance with underlying anatomical structures. Beyond established preclinical MRE applications, this methodology opens new avenues for multicenter biomechanical studies in small animal models and ex vivo specimens, with potential applications in oncology, regenerative medicine, and drug testing.

## Graphical abstract

MR elastography (MRE) noninvasively assesses tissue biomechanics, but preclinical research suffers from diverse hardware solutions, hindering reproducibility and comparability. This study introduces a versatile preclinical MRE device for imaging rodents, ex vivo samples, and spheroids/organoids. Validation, stability, and reproducibility experiments were conducted in phantoms and standard applications (rodent brain/abdomen). Furthermore, novel avenues such as the examination of ex vivo samples and spheroids/organoids were explored. By providing a standardized platform for rodent and specimen research, this device facilitates biomechanical preclinical studies.

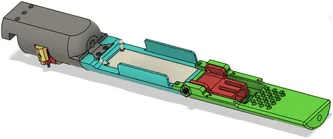

**Abbreviations**
AA: ambient air
alpha: shear absorption
BM: bone marrow
Cs: wave speed
|curl|/|div|: curl over divergence
CV: coefficient of variation
EM: electromagnetic
|G*|: magnitude of the complex shear modulus
MSC: mesenchymal stromal cells
SEM: standard error of mean
TTL: transistor–transistor logic
VOI: volume of interest

## 1 Introduction

Magnetic resonance elastography (MRE) is a noninvasive imaging technique for characterizing tissue biomechanics and microstructure, thereby providing information complementary to routinely used, predominantly structural magnetic resonance (MR) sequences [1]. Clinically, it is increasingly employed for the evaluation of liver fibrosis, and its potential clinical value for the diagnosis, characterization, and follow‐up of pathological conditions in various organs and tissues is studied by numerous research groups around the world [2, 3, 4, 5].

The advent of commercially available clinical MRE systems led to more uniformity regarding data acquisition and analysis. Furthermore, clinical studies have demonstrated promising results in liver fibrosis staging [6, 7] and have highlighted the potential of MRE in cancer characterization [2, 8]. However, in a clinical setting, histopathological analyses and their correlation with the observed tissue biomechanics remain limited.

To gain a deeper understanding of the cellular and structural mechanisms driving pathological changes, preclinical studies are indispensable. They enable extensive correlative histopathological analyses, highly controlled and reproducible experimental conditions, and higher spatial resolution, thereby providing fundamental insights into the relationship between tissue biomechanics and microstructure [9, 10, 11]. Yet, preclinical MRE suffers from the lack of commercially available, uniform hardware solutions resulting in a multitude of custom‐built devices utilized by different research groups [10, 12, 13, 14]. This hampers consistency and comparability between the results.

Furthermore, custom‐built devices are generally tailored to a single application, for example, rodent brain or liver [15, 16]. Next to this, the options for MRE quantification of in vitro tissue samples as well as spheroids and organoids are scarce given the difficulty of inducing proper shear waves inside these small samples.

The generic, multipurpose MRE device presented here, driven by an electromagnetic (EM) dual‐coil motor, addresses these current limitations of preclinical MRE. In this paper, we firstly demonstrate the system's frequency purity and its low motor heat dissipation. Thereafter, the reproducibility of data quality and measurement accuracy is demonstrated in gel phantoms as well as in rodent liver and brain applications, reflecting the most common use cases in the community. Lastly, we show the device's versatility and its potential use in novel MRE applications such as ex vivo tissue samples and spheroids.

## 2 Materials and Methods

### 2.1 Description of the Device

#### 2.1.1 Overall Design

The device is composed of three 3D‐printed main parts (Figure 1A).

1. A back frame harboring the dual EM coil motor (Figure 1A,B) and including a fixation unit at its very end for the attachment to the MRI table (here for Bruker small animal MRI scanners).
2. A middle frame featuring an integrated hot water circulation unit (temperature adjustable externally) which allows to maintain the animal's body temperature during rodent examination. In its inner body, it conceals a push–pull rod, which connects the dual EM motor with the transducer unit at the front frame (Figure 1C).
3. A front frame incorporating the vibrating transducer unit. The fixed outer unit of the front frame (green in Figure 1A) and the oscillating transducer unit (red in Figure 1A) are connected along a common axis, enabling controlled rotation of the transducer about this axis while preserving a robust mechanical pathway for efficient vibration transfer into the rodent or specimen. The transducer allows for the easy and precise attachment of different top units via two guiding rails.

**FIGURE 1:**
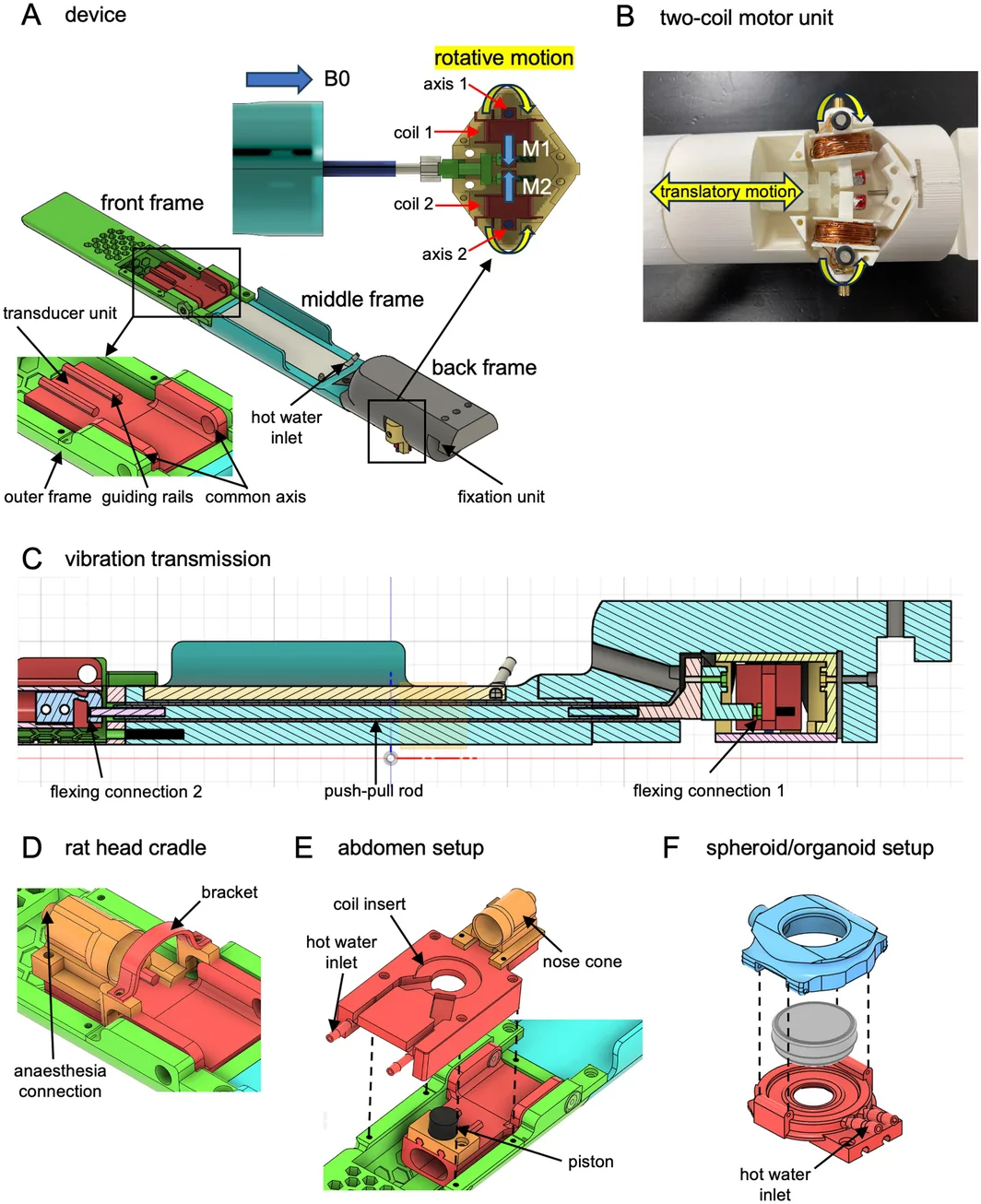
Illustration of the device and different top units. In (A), the device is displayed with its three main parts: the back frame harboring the EM motor, the middle frame with its integrated hot water tubing system, and the front frame harboring the transducer unit. The close‐up of the front frame (lower left corner) shows the axis which firmly connects the outer frame unit and the transducer unit. It further illustrates the two guiding rails used to position the various top units. A close‐up of the motor unit is also depicted in (A) (upper right corner) and in (B). The two coils are positioned in opposition to one another and orthogonally to B0 resulting in a total torque of the motor equal to zero when applying a sinusoidal current. The resulting translatory motion is transmitted from the first flexing connection along to the push–pull rod (concealed in the body of the middle frame) to the second flexing connection resulting in an up–down movement of the transducer unit (C). In (D), the rat head cradle is depicted with its nose cone to position the animal's muzzle, a bracket to allow firm positioning of the animal's head and its possible anesthesia tubing connection at the back. (E) illustrates the murine abdominal support with its central hole to allow the piston to transmit vibrations from the transducer unit to the animal's abdomen, the surrounding inlet for a surface coil, and a nose cone at its end allowing for anesthesia connection. (F) shows the examination support for spheroids and organoids which is directly fixated on the transducer unit and allows the examination of spheroids/organoids in a sealed petri dish.

#### 2.1.2 Dual EM Motor

The EM motor is composed of two current‐driven coils (Figure 1A,B). These coils are oriented within the MRI system's main magnetic field B0 such that their corresponding magnetic momenta are in opposition to one another and orthogonally oriented to B0. With the application of a sinusoidal current each coil oscillates around its corresponding axis leading to a total net torque of the motor that equals zero. In consequence, when operating within the main magnetic field B0, the motor will not turn or rotate around its own axis (Video S1).

#### 2.1.3 Vibration Transmission

The connection between EM motor and transducer is assured by a push–pull rod and two flexing connections (Figure 1C). The first flexing connection is mechanically linked to both coils and translates the opposed rotative motion of the oscillating coils into a translatory motion along the *z* direction which translates the push–pull rod (Figure 1B). The rod thus moves in pure translatory motion in the direction of B0 without any orthogonal motion components. Importantly, the coils operate in opposition; otherwise, the push–pull rod would not translate but simply be pushed sideways in an oscillatory manner. This requires that both coils operate in synchrony. The synchronicity is ensured by running the coils in parallel electrically with respect to the applied sinusoidal current. The second flexing connection translates the translatory back‐and‐forth motion of the push–pull rod into a rotative motion of the transducer leading hence to an up–down motion (Video S2).

#### 2.1.4 Top Units

Dedicated top units have been designed for different applications. Each top unit is tailored to optimize the acquisition conditions regarding signal‐to‐noise ratio (SNR) and ease of use while providing optimal transmission of shear waves into the object under investigation. Hence, we have designed for instance different head cradles for mouse and rat brain examination, and different examination supports for murine abdominal acquisitions and for ex vivo tissue specimens. All top units are 3D printed and can be quickly positioned on the vibrating transducer unit using its two guiding rails and/or the fixed outer frame (Figure 1A).

The head cradles dedicated for mouse and rat brain MRE each consist of a nose cone that securely positions the animal's muzzle, with an attachment point at the front end for the anesthesia tubing. They further feature a bracket that is fixated to two side blocks of the head cradle assuring thereby a firm and central positioning of the animal's head. The rat head cradle is shown as an example in Figure 1D.

To measure for instance murine liver biomechanics, the mouse is placed on a support positioned above the transducer (Figure 1E). The setup includes a system delivering anesthetic gas through a removable nose cone and a water‐circulating heating system integrated into the bed to maintain the animal's body temperature and prevent hypothermia. An opening in the bed allows the piston, mounted on the guiding rails of the transducer, to transmit vibrations in the mouse's abdomen ultimately inducing shear waves in the liver or other abdominal organs.

The examination unit for spheroids and organoids allows the fixation of a 35‐mm petri dish (Figure 1F), which in its entity is vibrating in the up/down direction. This concept has two advantages: Firstly, spheroids or organoids remain in a sealed petri dish and can be returned to standard culture conditions after the measurement. Secondly, this way of excitation evokes mainly planar shear waves that propagate from the bottom of the petri dish upwards which facilitates the quantification of biomechanical properties. Lastly, the integrated hot water tubing system, with externally adjustable water temperature, allows to stabilize the temperature at 37°C preventing thereby the sample's degradation (Figure 1F).

### 2.2 Motor Heat Dissipation Tests

To measure potential heat dissipation emitted by the system's EM motor, an MRI‐safe temperature probe (RTP‐103‐B, SA Instruments Inc., Stony Brook, NY, USA) was attached directly underneath the motor lid and temperature was monitored during three scans (one at 600 Hz and two at 1000 Hz) throughout 90 to 150 min using a monitoring and gating system (Model 1030, SA Instruments Inc.; Stony Brook, USA).

### 2.3 Accelerometer Tests

Parasite mechanical resonance frequencies generated by the EM motor and by the device itself would lead to artifacts in the data acquisition and hence impede the quality of the estimated biomechanical properties. Vibration measurements were performed directly inside the MRI scanner using a triaxial accelerometer (LSM6DSOX, 6.7‐kHz sampling frequency, Adafruit, New York, USA) fixed to the transducer unit by means of a dedicated top unit. The accelerometer chip was read out via a Raspberry Pi (Raspberry Pi 3 Model B+, Cambridge, UK). Sinusoidal vibrations were applied at frequencies ranging from 250 to 1800 Hz, in 50‐ or 100‐Hz increments. For each frequency, acceleration signals were recorded over a 12‐s period, capturing the time‐domain response along the three axes (*x*, *y*, and *z*) and revealing the dominant oscillatory component. The data, expressed in g, were then transformed into the frequency domain, highlighting the amplitude distribution across frequencies. In theory, a sharp and isolated peak at the excitation frequency indicates a stable and well‐defined vibration.

### 2.4 Phantom Tests for Assessing Reproducibility

Reproducibility of data acquisitions was assessed using phantoms made from ultrasound gel (Anagel, Surrey, UK). Experiments were conducted using a petri dish filled with approximately 10 mL of gel placed into the spheroid/organoid top unit setup.

### 2.5 Regulatory Aspects and Animal Handling

All animal experiments were approved by the French governmental authorities (Animal Protocol # 51789‐2024101411059997) and performed in compliance with the laboratory animal research guidelines.

Mice (C57BL6, *n* = 3, male) and rats (Wistar, *n* = 2, male) were purchased from Janvier Labs (Le Genest‐Saint‐Isle, France) at a minimum age of 8 weeks and first used for experiments 2 weeks later. They were housed in their respective specific pathogen‐free animal facility of the Faculté de Médecine Xavier Bichat, Paris, France (Agreement Number D751801) with a 12/12‐h light/dark cycle and food and water ad libitum.

### 2.6 Specimen and Cell Preparation

Chicken thighs (*n* = 5) were freshly purchased at a local butcher shop. Specifically, the sciatic nerve was dissected, swiftly washed in phosphate‐buffered saline (PBS, Gibco, Ref. # 12549079) and then embedded in 1% agar gel (Sigma‐Aldrich, Ref. # A9539). Between the two performed scans (on Days 1 and 6 post dissection), the samples were stored at 4°C.

A turkey skin sample with its subjacent tissue layers (*n* = 1) was collected from a local butcher shop and scanned the same day. To prevent dehydration during scanning, it was wrapped in cellophane.

Regarding spheroid preparation, bone marrow (BM) samples were obtained from patients without known hematological disease who were admitted to the Orthopedic and Trauma Surgery Department of Lariboisière Hospital (Paris, France) for total hip replacement surgery. All patients provided written informed consent in accordance with the Helsinki Declaration. Approval from the Ethics Review Committee for Biomedical Research Projects Paris Nord (CEERB, IRB # 00006477) was obtained prior to the use of clinical materials for research purposes.

Mononuclear cells were purified from the BM using a Ficoll gradient (Pancoll Human, PAN‐BIOTECH, Ref. # 17130949). Human mesenchymal stromal cells (h‐MSCs) were derived from BM samples and separated from other hematopoietic cells based on their plastic adherence after 2–3 weeks of in vitro culture. The h‐MSCs were then expanded in αMEM (Gibco, Ref. # 32561‐029) supplemented with 10% fetal bovine serum (FBS, Sigma Eurobio, Ref. # CVFSVF00‐01), 2‐mM glutamine, 100‐U/mL penicillin, and 100‐μg/mL streptomycin (Gibco, Ref. # 15140122). When the h‐MSCs reached 80%–90% confluence, the cells were detached with trypsin (Gibco, Ref. # 15400054) and cryopreserved before use [17].

For imaging, individual spheroids (*n* = 3) were embedded in a dome of type I rat tail collagen of 3 mg/mL (Corning, Ref. # 354249) supplemented with 200‐mM d‐ribose (ThermoScientific, Ref. # A17894.18) to accelerate gel crosslinking and enhance matrix stiffness as previously described [18]. This collagen‐based hydrogel has been shown to provide a biocompatible and mechanically stable environment that preserved spheroid viability while enabling efficient propagation of shear waves during MRE acquisition [19].

### 2.7 MRE Data Acquisition

MRE data acquisition was performed using a 7‐T Bruker small animal MRI scanner (BioSpec 70/30 USR, Bruker BioSpin GmbH, Ettlingen, Germany; gradient strength 660 mT/m) in conjunction with a ^1^H transmit–receive volume coil with active detuning (86 mm of inner diameter) and ^1^H‐planar receive‐only surface coils (20 and 30 mm of inner diameter). However, compatibility with other horizontal MRI scanners, such as the Bruker PharmaScan 7 T and the Bruker BioSpec 94/20 USR featuring the AutoPac positioning system (both Bruker BioSpin GmbH, Ettlingen, Germany), was also successfully tested.

A function generator, triggered by TTL pulses from the MRI scanner, was used to drive the EM motor at a specified frequency, thereby ensuring synchronization between mechanical vibration and image acquisition. MRE acquisition consisted of a multislice multiecho (MSME) sequence with a single echo. Note that the MRE‐sequence parameters and vibration frequencies were adapted to suit the examined specimen or organ/tissue. Parameter details of the employed sequences are provided in Table 1.

**TABLE 1:** Employed sequences and their parameters.

Sequence	TE (in ms)	TR (in ms)	Averages	Acquisition matrix (in mm)	FoV (in mm)	Slice thickness (in mm)	Number of slices	Acquisition time
3D MSME 250‐Hz US gel	24	1200	1	64 × 64	35 × 35	0.5	15	20 min 30 s
3D MSME 1000‐Hz US gel	27	1200	1	128 × 128	36 × 36	0.28	15	41 min
3D MSME 1400‐Hz US gel	30.7	1200	1	128 × 128	36 × 36	0.28	15	41 min
3D MSME, 500‐Hz rat brain	24	1200	1	64 × 64	35 × 35	0.5	15	20 min 30 s
3D MSME, 750‐Hz mouse brain	24	1200	1	64 × 64	19.2 × 19.2	0.3	15	20 min 30 s
3D MSME 250‐Hz mouse liver	16	840	2	64 × 64	48 × 48	1	15	29 min
2D MSME, 1000‐Hz nerves	24	1200	4	64 × 64	19,2 × 19,2	0.3	15	1 h 01 min
1000‐Hz organoids/spheroids	26	915	4	512 × 128	18 × 36	0.5	15	2 h 04 min
1000‐Hz Turkey skin	29	570	4	256 × 128	36 × 36	0.5	15	1 h 04 min

Abbreviations: FoV = field of view, MSME = multislice multiecho, TE = echo time, TR = repetition time.

For rodent MRI scans, anesthesia was induced with 1.5%–3.0% isoflurane (ISO‐VET, Paris, France) in 50% O_2_/50% ambient air (AA) and maintained during the scan with 1.0%–3.0% isoflurane in 50% O_2_/50% AA. The animal's respiration rate was monitored throughout the scan via a surface pad and a monitoring and gating system (Model 1030, SA Instruments Inc.; Stony Brook, USA). To assure the stability of the rodents' body temperature, the circulating hot water system of the device's middle frame was employed.

Embedded chicken nerves were positioned on a dedicated top unit and scanned on Days 1 and 6 post dissection.

The examination of a turkey skin sample with its subjacent tissue was performed by fixing the sample directly on a piston, the latter being positioned on the transducer unit's guiding rails.

Spheroids were scanned 3 days after seeding and 1 day after embedding in the collagen matrix. To prevent degradation and cell death during MR‐acquisition, a hot water circulation system integrated in the dedicated top unit was used and the maintenance of the sample's temperature at 37°C was monitored with an MRI‐safe temperature probe attached beneath the petri dish.

### 2.8 MRE Data Postprocessing and Analysis

Reconstruction and analysis of MRE data was performed according to published algorithms using dedicated in‐house software [20]. Succinctly, per slice unwrapping of phase data and then alignment between slices was performed. As four wave phases were acquired, a 4‐point Fourier transform was calculated, and the first harmonic amplitude was employed to represent the displacement amplitude and its phase, constituting the phasor components of the steady‐state solution of the 3D wave propagation. The curl operator was applied on the displacement data to remove the compressional component of the waves. Calculation of spatial derivatives was performed and used to invert the Helmholtz equation [21].

Quality control markers adapted from Koch et al. were applied to assess data quality [20]. Specifically, the total amplitude of the induced mechanical waves (*A*_tot_ in μm) and the nonlinearity (i.e., the % deviation from a perfect sinus at the main carrier frequency quantified through the presence of energy in the upper harmonics) were evaluated for each data set. The ratio of the curl over the divergence (|curl|/|div|) of the 3D motion field was additionally assessed for isotropic 3D sequences, as it represents the SNR of the shear wave field.

For the ultrasound gel phantom, the magnitude of the complex shear modulus |G*| (kPa), the wave speed Cs (m/s), the shear absorption alpha (1/m), and the phase angle *Y* (*π*/2) were determined. |G*|, also referred to as “stiffness,” is composed of an elastic (shear modulus *G*_d_) and viscous part (loss modulus *G*_l_) and is calculated via $\left|G*\right|=\sqrt{{G_{d}}^{2}+{G_{l}}^{2}}$. The phase angle expresses the relative contributions of loss to shear modulus and is defined via $Y=\frac{2}{\pi }\;\text{atan}\left(\frac{G_{l}}{G_{d}}\right)$. Thus, for a purely elastic material, *Y* = 0 and for a purely viscous material *Y* = 1.

For rodents and tissue samples, MRE parameters are displayed in color‐coded maps (elastograms).

For mouse brain, the cortex (from 1‐mm rostral to 1‐mm caudal of the bregma) was segmented on eight consecutive slices. |G*|, *Y*, Cs, and alpha were determined for these volumes of interest (VOIs) in three mice on three consecutive scans for each animal.

For mouse liver, the same biomechanical parameters were extracted in three mice on three consecutive scans for each animal. Four to six consecutive slices were selected to define the VOIs.

As nerves were examined by 2D‐MRE using directional filtering for reconstruction [22], only |G*| was calculated for these samples.

For turkey skin and the subjacent tissue layers, the propagation of the shear wave was visualized to assess the feasibility of layer‐dependent analysis. A line was drawn across three central slices, spanning the skin layers and underlying tissue, to display the variation of the wave pattern from the rotational component in *x* direction (*Q*_*x*_).

For MSC spheroids, the complex displacement field was analyzed along a line passing through the center of the sample to extract the local wave profile. The displacement is represented as a complex‐valued wave function with a real and an imaginary part, corresponding to two components of the same oscillation shifted by 90° in phase. We utilize a simultaneous 1D fitting of an attenuated sinus function to the real and imaginary part under the assumption that amplitude, phase, and wave number are the same for both components. This approach provides a more reliable estimation of the local wavelength and thus shear wave speed. This 1D approach for estimating shear wave properties is utilized specifically for this application as image resolution is highly anisotropic: While the spheroid is spatially resolved at 35 μm along the propagation direction of the quasiplanar shear wave, the other two image resolutions are 280 and 500 μm. Isotropic image resolution at 35 μm allowing for a proper 3D inversion would lead to prohibitive long data acquisition times making this simplification an experimental necessity.

### 2.9 Statistical Analysis

Statistical analysis was performed with descriptive statistics and where applicable paired *t*‐tests were conducted (Prism version 10.6.0; GraphPad Software Inc., La Jolla, CA, USA). Values are expressed as mean ± standard error of mean (SEM), a *p* value ≤ 0.05 was considered significant. Further parameters, like maximum discrepancies between different acquisitions (range) and the coefficient of variation (CV) were assessed.

## 3 Results

### 3.1 System and Frequency Stability and Reproducibility

#### 3.1.1 Motor Heat Emission

For the three acquired scans, lasting between 90 and 150 min, mean temperatures of 20.03°C ± 0.19°C (Scan 1), 21.86°C ± 0.11°C (Scan 2), and 20.84°C ± 0.15°C (Scan 3) were recorded, with maximum variations of 2.49°C, 1.00°C, and 2.00°C, respectively. These results demonstrate negligible heat dissipation and minimal temperature increase, even during prolonged acquisitions (Figure 2A).

**FIGURE 2:**
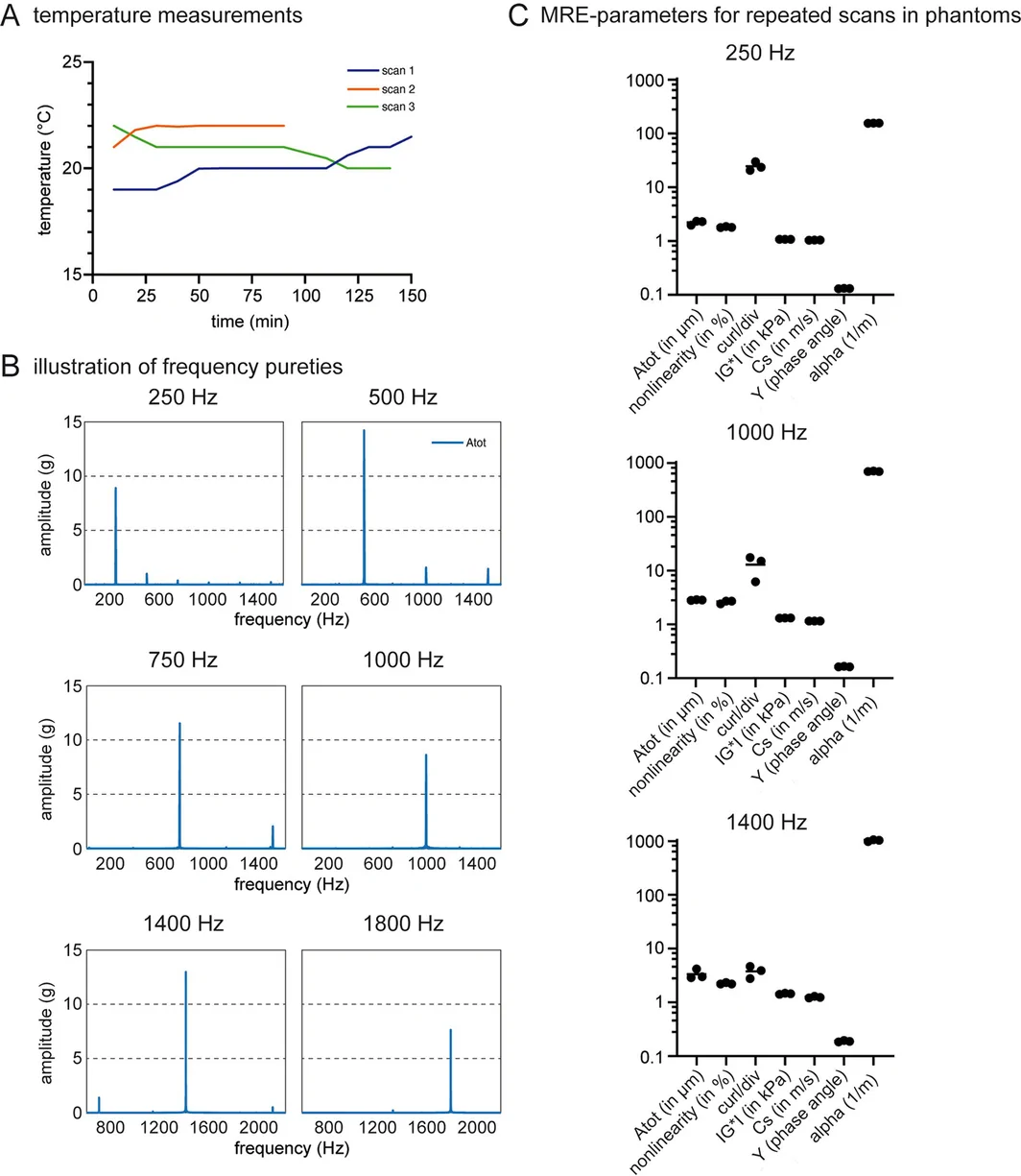
Evaluation of the device's heat emission, frequency purity, and reproducibility. In (A), three temperature curves (monitored by the means of a sonde attached to the motor unit) are shown. For acquisitions ranging between 90 and 150 min in length a maximum temperature variation of 2.49°C was observed (Scan 1) with mean temperatures of 20.03°C ± 0.19°C (Scan 1), 21.86°C ± 0.11°C (Scan 2) and 20.84°C ± 0.15°C (Scan 3). Spectra of frequency purity are depicted in (B) for 250, 500, 750, 1000, 1400, and 1800 Hz revealing a dominant peak at their given excitation frequency with minor upper second harmonics (maximum interference of 17.9% at 700 Hz for the 1400 Hz acquisition). (C) depicts the scan quality (*A*_tot_, nonlinearity, and |curl|/|div|) and the retrieved values for Cs, *Y*, |G*|, and alpha in ultrasound gel phantom scanned three consecutive times at 250, 1000, and 1400 Hz revealing a high reproducibility between the acquisitions. Data is presented in log10 scaling.

#### 3.1.2 Frequency Stability

Frequency spectra at driving frequencies ranging from 250 to 1800 Hz showed a single, dominant peak at their respective excitation frequency with the presence of small upper second harmonics (11.2% at 250 Hz, 11% at 500 Hz, 17.7% at 750 Hz, 10% at 1000 Hz, 17.9% at 1400 Hz, and 3.3% at 1800 Hz). The spectra for 250, 500, 750, 1000, 1400, and 1800 Hz are depicted in Figure 2B. The presence of harmonics below 20% of the main peak confirms the spectral purity of the generated mechanical vibrations.

#### 3.1.3 Reproducibility in Ultrasound Gel Phantoms

Figure 2C shows the results of data quality and reproducibility obtained from three consecutive MRE scans at 250‐, 1000‐, and 1400‐Hz vibration frequency using the ultrasound gel phantom (Table 2). Nonlinearity was consistently lower than 5%, while |curl|/|div| values were above 3. Furthermore, for a given frequency, shear waves of constant amplitude were induced in the phantoms (maximum range of 0.37 μm for 250 Hz, 0.08 μm for 1000 Hz, and 1.29 μm for 1400 Hz), highlighting the system's stability. This constitutes the necessary foundation for obtaining highly reproducible biomechanical data with little variance and a CV below 5% (Table 3).

**TABLE 2:** Quality control markers of acquired data for ultrasound gel phantoms and mouse brain and liver.

Model	Frequency (Hz)	*A*_tot_ (mean ± SEM) (μm)	*A*_tot_ range (μm)	*A*_tot_ CV (%)	Nonlinearity (mean ± SEM) (%)	Nonlinearity range (%)	Nonlinearity CV (%)	curl/div (mean ± SEM)	curl/div range	curl/div CV (%)
Ultrasound gel	250	2.21 ± 0.12	0.37	9.25	1.81 ± 0.03	0.09	2.67	24.72 ± 2.78	9.40	19.45
Ultrasound gel	1000	2.84 ± 0.02	0.08	1.36	2.61 ± 0.10	0.31	6.82	12.94 ± 3.44	11.37	46.10
Ultrasound gel	1400	3.35 ± 0.41	1.29	21.36	2.25 ± 0.04	0.13	3.28	3.77 ± 0.55	1.9	25.28
Brain Mouse 1	750	2.13 ± 0.088	0.30	7.16	3.87 ± 0.34	1.10	15.15	8.38 ± 0.24	0.78	4.85
Brain Mouse 2	750	2.43 ± 0.18	0.60	12.56	2.77 ± 0.67	2.10	41.89	9.4 ± 0.33	1.14	6.07
Brain Mouse 3	750	2.2 ± 0.01	0.01	0.01	4.47 ± 0.34	1.10	13.12	8.95 ± 0.11	0.39	2.18
Liver Mouse 1	250	9.88 ± 0.91	3.06	15.95	23.18 ± 3.54	12.00	26.42	4.36 ± 0.73	2.42	28.87
Liver Mouse 2	250	6.88 ± 0.77	2.31	19.3	21.3 ± 2.45	8.31	19.90	3.63 ± 0.92	2.94	43.83
Liver Mouse 3	250	6.46 ± 0.29	0.93	7.78	24.18 ± 4.52	14.61	32.40	5.25 ± 1.16	4.00	38.17

Abbreviations: *A*_tot_ = total amplitude of induced mechanical waves, curl/div = curl over divergence, CV = coefficient of variation, range = max–min.

**TABLE 3:** Biomechanical characteristics of ultrasound gel phantoms and mouse brain and liver.

Model	Frequency (Hz)	Cs (mean ± SEM) (m/s)	Cs range (m/s)	Cs CV (%)	|G*| (mean ± SEM) (kPa)	|G*| range (kPa)	|G*| CV (%)	*Y* (mean ± SEM)	*Y* range	*Y* CV (%)	Alpha (mean ± SEM) (1/m)	Alpha range (1/m)	Alpha CV (%)
Ultrasound gel	250	1.04 ± 0.01	0.01	0.25	1.08 ± 0.01	0.01	0.39	0.13 ± 0.01	0.01	0.88	156 ± 0.44	1.50	0.48
Ultrasound gel	1000	1.16 ± 0.01	0.01	0.13	1.31 ± 0.01	0.01	0.23	0.16 ± 0.01	0.01	1.33	702 ± 5.23	15.80	1.29
Ultrasound gel	1400	1.25 ± 0.03	0.09	3.51	1.45 ± 0.02	0.07	2.46	0.19 ± 0.01	0.01	3.07	1027 ± 23.03	77.40	3.88
Brain Mouse 1	750	2.3 ± 0.058	0.20	4.35	4.7 ± 0.15	0.50	5.63	0.39 ± 0.01	0.03	3.95	657 ± 18.4	57.50	4.84
Brain Mouse 2	750	2.37 ± 0.067	0.20	4.88	5.0 ± 0.15	0.50	5.29	0.39 ± 0.01	0.01	1.47	642 ± 5.8	18.00	1.55
Brain Mouse 3	750	2.33 ± 0.033	0.10	2.47	4.97 ± 0.03	0.10	1.60	0.37 ± 0.01	0.02	2.70	596 ± 20.8	72.10	6.05
Liver Mouse 1	250	1.41 ± 0.039	0.14	4.83	1.92 ± 0.1	0.31	8.20	0.25 ± 0.02	0.08	15.99	219 ± 17	54.30	13.47
Liver Mouse 2	250	1.41 ± 0.029	0.09	3.55	1.87 ± 0.1	0.29	8.17	0.28 ± 0.01	0.03	4.95	243 ± 10.3	31.60	7.33
Liver Mouse 3	250	1.45 ± 0.064	0.22	7.60	2.07 ± 0.2	0.63	15.57	0.20 ± 0.02	0.07	20.04	171 ± 18.8	59.60	19.02

Abbreviations: Alpha = shear absorption, Cs = wave speed, CV = coefficient of variation, |G*| = complex shear modulus, range = max–min, *Y* = phase angle.

As expected, the increase in excitation frequency from 250 to 1000 Hz and further to 1400 Hz was biomechanically reflected in an increase of Cs (1.04 ± 0.01 m/s at 250 Hz, 1.16 ± 0.01 m/s at 1000 Hz, and 1.25 ± 0.03 m/s at 1400 Hz), |G*| (1.08 ± 0.01 kPa at 250 Hz, 1.31 ± 0.01 kPa at 1000 Hz, and 1.45 ± 0.02 kPa at 1400 Hz), alpha (156 ± 1.50 1/m at 250 Hz, 702 ± 5.23 1/m at 1000 Hz, and 1027 ± 23.03 1/m at 1400 Hz), and the phase angle *Y* (0.13 ± 0.01 at 250 Hz, 0.16 ± 0.01 at 1000 Hz, and 0.19 ± 0.01 at 1400 Hz).

### 3.2 Rodent Applications

MRE acquisitions in mouse brains were performed at frequencies ranging from 500 to 875 Hz and in rat brains ranging from 250 to 625 Hz. The resulting elastograms allowed the clear discrimination of different expected anatomical substructures such as the corpus callosum or the basal ganglia and revealed high symmetry between the right and left hemisphere as well as reproducibility between different acquisitions (Figure 3A,B).

**FIGURE 3:**
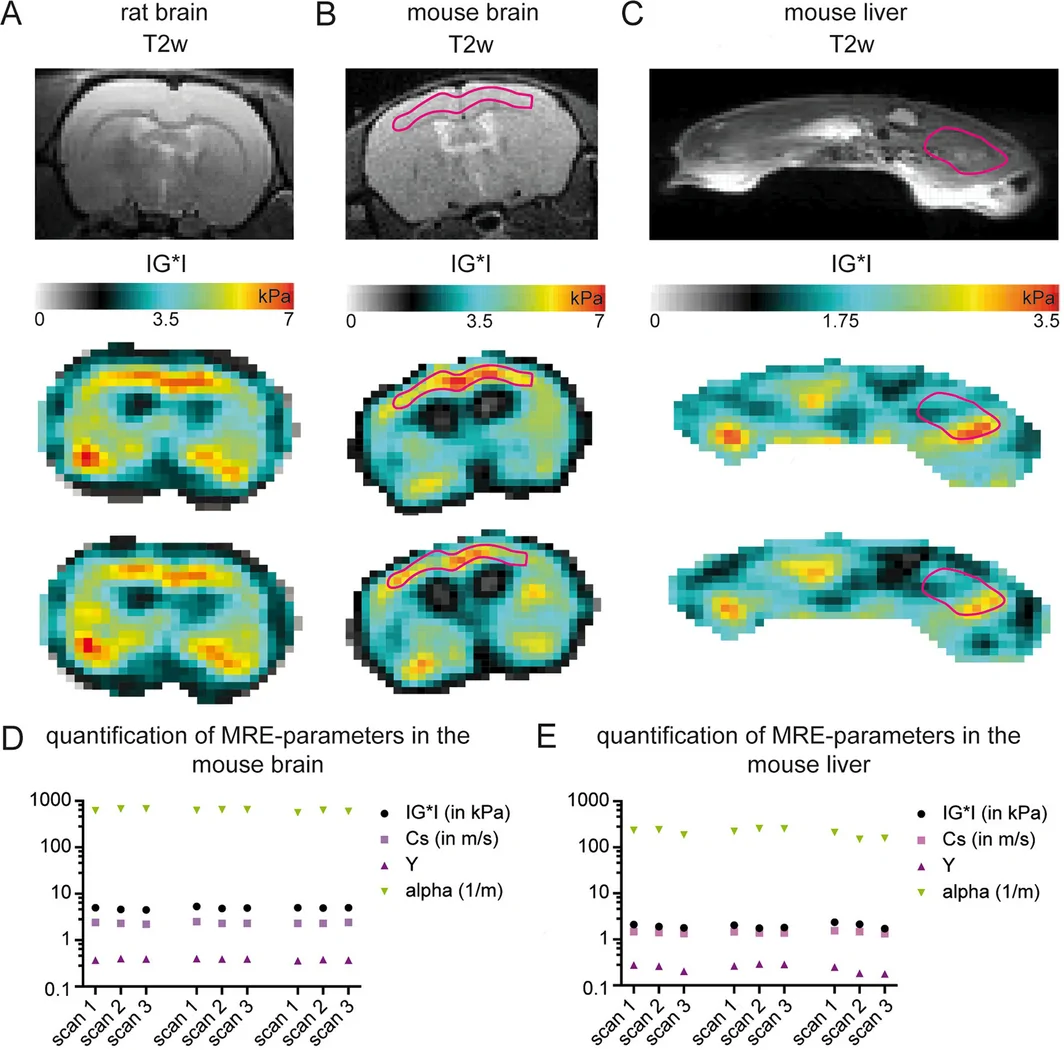
Examples of the device's in vivo applications. In (A), |G*| elastograms of repeated scans (acquired at 500 Hz) of a rat brain with its corresponding T2w image are depicted. (B) shows |G*| elastograms (acquired at 750 Hz) and the T2w image of a mouse brain. In both cases, high symmetry between the two hemispheres and delineation of anatomical structures such as the ventricles can be observed. The |G*| elastograms and T2w image of a mouse abdomen are shown in (C). In (D), |G*|, Cs, *Y*, and alpha of three mice undergoing three consecutive scans are quantified, and a VOI placed in the cortex stretching from −1 to +1 mm of the bregma was used. In (E), |G*|, Cs, *Y*, and alpha of a VOI placed in the left liver lobe are quantified (also using three mice, each undergoing three consecutive scans). In both cases, high reproducibility of biomechanical parameters was achieved. Data are displayed in log10 scaling.

High‐quality data and reproducibility over three scans could be observed with a nonlinearity constantly below 5% and a |curl|/|div| above 5. The amplitude of the induced shear waves was also highly stable with a maximum range of 0.6 μm and a CV of 12.56% observed for Mouse 2 (Table 2).

Segmented mouse cortex (±1 mm of the bregma) showed highly reproducible biomechanical parameters as presented in Figure 3D and Table 3.

Considering the inherent motion limitations in hepatic imaging due to breathing and adjacent bowel movements mouse liver experiments performed at frequencies ranging from 250 to 325 Hz demonstrated satisfactory data quality with a nonlinearity constantly below 30% and a |curl|/|div| above 2.5 as well as a maximum *A*_tot_ range of 3.06 μm and a CV of 15.95% (Mouse 1, Table 2). Thereby highly stable biomechanical parameters could be obtained in a defined VOI in the right liver (Figure 3C,E and Table 3).

### 3.3 Ex Vivo Specimen Applications

As of now, application of MRE in small ex vivo specimens is scarce [23, 24]. Given the achieved high data quality and reproducibility in common applications (phantoms and rodents), we also investigated the feasibility to use this device in novel applications such as ex vivo tissue samples (nerve and skin) and spheroids.

Scans of embedded chicken nerves provided excellent data quality with maximum nonlinearity of 5.92% (Sample 4 on Day 1). Visually, the nerves could be identified easily on elastograms and revealed a mean |G*| of 29.68 ± 1.34 kPa on Day 1. Rescanning of the nerves on Day 6 post extraction showed sensitivity to temporal decay with a significantly lower |G*| of 26.06 ± 0.99 kPa (Figure 4A,B). This finding is in accordance with other studies that found—after an initial increase in stiffness—a decay in postmortem tissue stiffness over time in different organs and species [25, 26].

**FIGURE 4:**
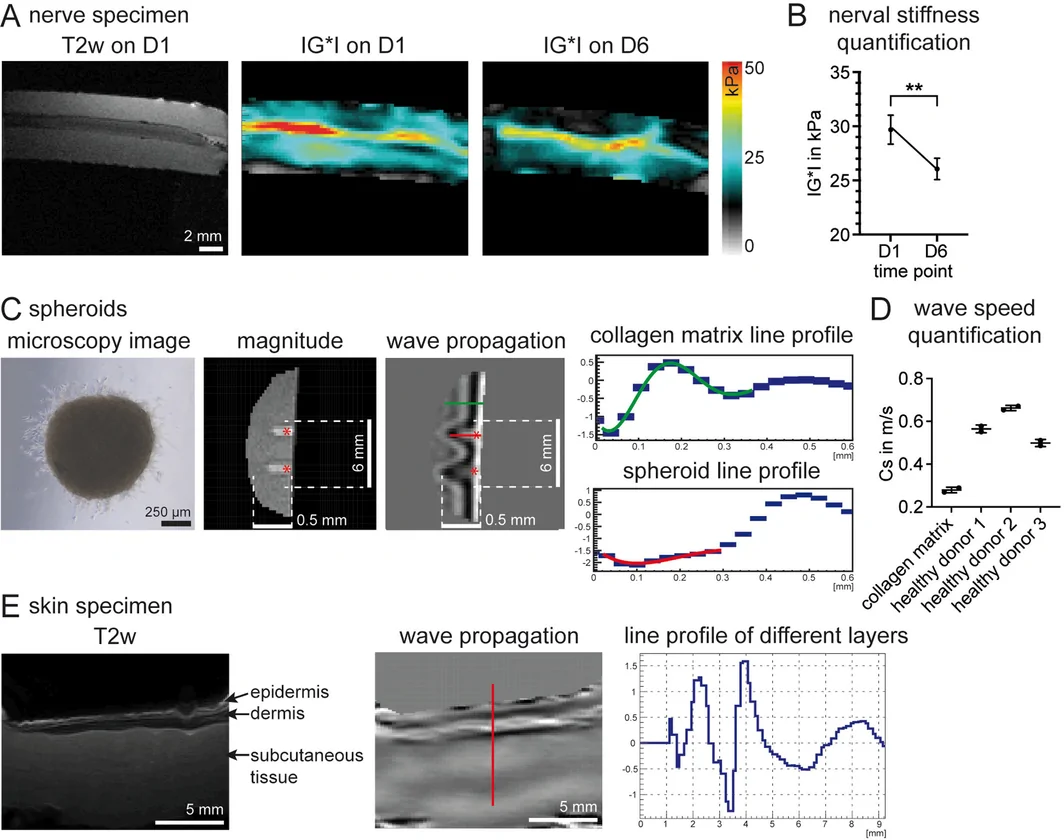
Examples of the device's ex vivo*/*in vitro applications. (A) shows the T2w image and the |G*| elastograms of a dissected chicken sciatic nerve on Days 1 and 6 post dissection. At both time points, the nerve was easily delineated on the elastograms as a markedly stiff structure. The nerval stiffness |G*| is quantified in (B) with a mean of 29.68 ± 1.34 kPa on Day 1 and of 26.06 ± 0.99 kPa on Day 6. In (C), a microscopy image of a MSC spheroid is shown alongside with a magnitude and wave propagation image of two spheroids (indicated by the red asterisks). Line profiles of the collagen matrix and the upper spheroid (extracted respectively from the green and red line in the wave propagation image) are also depicted. In (D), the wave speed of three MSC spheroids (ranging between 0.50 ± 0.01 and 0.66 ± 0.01 m/s) and the collagen matrix (0.28 ± 0.01 m/s) is quantified. Note the possibility to distinguish the spheroids from their background matrix visually and quantitatively. (E) shows a turkey skin specimen with its underlying subcutaneous tissue in T2w with its corresponding wave propagation image and the resulting line profile (extracted from the red line in the wave propagation image). Different wave speeds can be discerned in the different layers.

The MSC spheroids displayed good data quality with a maximum nonlinearity of 13.29% (Sample 3). The surrounding collagen matrix revealed a nonlinearity of 18.9%. Markedly higher wave speeds were observed in the spheroids (MSC Donor 1 Cs = 0.57 ± 0.01 m/s, MSC Donor 2 Cs = 0.66 ± 0.01 m/s, and MSC Donor 3 Cs = 0.50 ± 0.01 m/s) compared with the surrounding collagen matrix (Cs = 0.28 ± 0.01 m/s, Figure 4C,D).

In the turkey skin sample, three distinct layers could be identified on the T2‐weighted image (Figure 4E). Across these layers, a mean nonlinearity of 8.28% was retrieved. The superficial hyperintense layer corresponded to the epidermis. Beneath it, a dark intermediate layer was visible, representing the dermis. The deepest layer with intermediate to high signal intensity corresponded to the underlying subcutaneous tissue from the thigh region. The resulting displacement profile showed a clear variation of wavelength across the different tissue layers, with noticeably longer wavelengths observed in the muscle compared with the overlying skin regions indicative of its elevated stiffness (Figure 4E). This qualitative observation highlights the ability of the present setup to capture wave propagation through structurally and mechanically heterogeneous biological tissues.

## 4 Discussion

Here, we present a novel preclinical MRE device that distinguishes itself from existing devices by its double‐coil EM motor and its versatility in serving multiple applications ranging from in vivo to ex vivo.

The two‐coil EM actuator offers several advantages over commonly used piezoelectric, electroacoustic, and off‐the‐shelf EM actuators (e.g., Hottinger Brüel & Kjær Mini Shaker Type 4810). A major limitation of piezoelectric and conventional EM actuators is their heat emission [16], whereas our dual‐coil design exhibited only minimal temperature variations, not exceeding 2.5°C, and remained quasi at ambient temperature. In addition, piezoelectric actuators are typically cost‐intensive [27], in contrast to the low manufacturing cost of our 3D‐printed device, including the integrated EM coils.

In general, MR image quality can be impacted by the residual EM‐stray field of classical actuators resulting in detrimental image artifacts for MRE [16]. On the contrary, our double‐coil approach leads to an intrinsic annihilation of the EM field on the *z* axis in left–right direction thus minimizing stray field artifacts.

Electroacoustic actuators usually perform poorly regarding high‐frequency transmission given their inherent design and suffer frequency inaccuracy [27]. Our device, in contrast, showed highly reproducible data even at excitation frequencies up to 1400 Hz. Spectra were highly accurate at the expected driving frequency with small second harmonics and no side bands, that is, parasite resonance frequencies. Furthermore, high‐quality spectra at frequencies up to 1800 Hz were acquired showing the device's potential for acquisitions at driving frequencies in samples demanding higher resolution. Although the device itself can operate at high frequencies, acquisitions exceeding 2000 Hz are currently impractical on standard preclinical MRI systems because of gradient duty‐cycle limitations and the prohibitively long acquisition times that would otherwise be required.

Reproducibility tests performed in ultrasound gels yielded high‐quality data and demonstrated a high degree of consistency between scans. For instance, the shear wave speed was 1.04 ± 0.01 m/s at 250 Hz, 1.16 ± 0.01 m/s at 1000 Hz, and 1.25 ± 0.01 m/s at 1400 Hz. This increase in Cs reflects the biomechanically expected dispersion at different driving frequencies. Furthermore, these values fall within the range of what has previously been reported in the literature using various preclinical setups. For context, previous studies reported 0.95 m/s at 500 Hz on a preclinical 7‐T scanner [28], 0.77 ± 0.09 m/s using a multifrequency approach (1000–1400 Hz) [23] on a preclinical 7‐T scanner, and 1.3 ± 0.1 m/s using a tabletop MRE system at 0.5 T with a multifrequency approach (400–2000 Hz) [29]. The discrepancies between our results and the latter two studies likely stem from our monofrequency focus versus their multifrequency approaches, which combine data across several frequencies.

Like piezo‐based motors, our dual‐coil motor can be positioned very close to the transducer, avoiding the need for a long transmission axis. This feature represents a valuable advantage of our device: It is compact, very easy to position, and can be handled easily by one person. While some systems rely on a relatively long flexible transmission rod, our design incorporates a rather short rigid rod (~30 cm) that is sliding within a low‐friction hollow tube made from poly‐acryl, which is rigidly anchored to the middle frame. This compact configuration reduces the likelihood of any mechanical buckling of the rod, resulting in a reliable wave transmission with minimal upper/lower harmonics generated by the push–pull rod.

The setup has been primarily evaluated in a Bruker BioSpec 7 T. However, it has also been tested and used in a Bruker 7‐T PharmaScan and a Bruker BioSpec 9.4 T, highlighting the potential for more uniform data acquisition between different scanners, centers, and research groups.

Furthermore, the MRE postprocessing software pipeline is similar in terms of physics to one of the clinical elastography systems, thereby bridging the gap between preclinical and clinical research and facilitating data comparisons.

Another advantage of our device is its versatility and hence its possible use in a multitude of applications. To the best of our knowledge, most reported devices are tailored to the use of a single preclinical application. Our device, with its easily exchangeable top units, allows the examination of various samples ranging from in vivo rodent imaging to tissue samples and even organoids or spheroids.

One of the most widespread applications of MRE in preclinical research is the investigation of rodent brain biomechanics. Here, our setup demonstrates highly reliable data acquisition, as evidenced by low nonlinearity values, high curl‐to‐divergence ratios, and consistently high $A_{\mathrm{tot}}$ values with minimal variation across scans. Collectively taken, these metrics provide a quantitative assessment of data quality and wave field integrity that are not consistently observed in previously published preclinical brain studies.

Hepatic MRE acquisitions exhibited lower data quality compared with phantoms, rodent brain, and ex vivo specimen. One must bear in mind that liver imaging is naturally impeded by motion artifacts stemming from breathing and bowel movements. Consequently, this results in greater physiological variability in the retrieved biomechanical properties, which must be taken into account when studying hepatic, and more broadly abdominal, pathology in rodents. Motion‐gated acquisition protocols may help mitigate these effects.

Our device allows the investigation of the biomechanics in models that, to our knowledge, have never been explored using MRE, such as spheroids or ex vivo nerves and skin.

Compared with “bulk tissue” (e.g., ex vivo specimen), the shear wave speeds measured in the spheroid samples were low in absolute terms, with values of 0.50–0.66 m/s in MSC spheroids and 0.28 ± 0.01 m/s in the surrounding collagen matrix. However, these values are consistent with measurements performed in small, highly hydrated, cell‐compatible collagen‐based constructs. The collagen matrix value corresponds to an apparent shear modulus of approximately 80 Pa, while the spheroid values correspond to approximately 250–435 Pa, assuming a density close to that of water. Thus, the spheroids exhibited higher stiffness than the surrounding collagen matrix.

The collagen matrix value was slightly lower than previously reported MRE‐derived values for untreated 3‐mg/mL collagen gels, for which storage moduli of approximately 100–200 Pa have been reported by Sauer et al. [30]. This difference may be explained by methodological differences, including collagen source, preparation protocol, sample geometry, and the presence of excess culture medium during overnight incubation and MRE acquisition. Therefore, these values should be interpreted as apparent shear wave speeds measured under cell‐compatible experimental conditions rather than as direct equivalents of bulk tissue stiffness.

These examples highlight the wide range of experimental configurations that can be addressed and demonstrate the adaptability of our approach to diverse biological systems. This versatility opens new avenues for applications in both fundamental research and preclinical studies and may help extend the use of MRE into domains that remain largely unexplored. In organoid models for instance, MRE could provide a noninvasive read‐out of tissue maturation or treatment response, complementing molecular and structural assays while allowing longitudinal monitoring of the same sample. It furthermore opens the avenue to investigate treatment response using patient‐derived tissue samples for selecting the optimal drug with the ability to utilize the same imaging biomarkers in preclinical and clinical imaging.

Future sequence developments will focus on the integration of structural and other quantitative MR techniques (such as DTI or T1/T2 relaxometry). The device's possible integration in clinical MRI systems operating at 1.5 and 3 T will be soon explored given the systems nonmagnetic properties and its operability in any magnetic field through the specific EM motor design.

Collectively taken, our device offers, compared with existing setups, enhanced compatibility with a variety of preclinical MRI scanners and the possibility to examine diverse small biological samples with high data quality at a low manufacturing cost.

## 5 Conclusion

This study introduces a dedicated MRE device for preclinical research, addressing the limitations of current systems. Its successful validation on phantoms, ex vivo tissues, and rodents underscores its potential for advancing biomechanical investigations in preclinical models.

## Author Contributions

Ralph Sinkus and Katharina Schregel had the idea for the study. Gabrielle Mangin, Hannah Fels‐Palesandro, Katharina Schregel, and Ralph Sinkus designed all experiments. Gabrielle Mangin, Hannah Fels‐Palesandro, Marguerite Ducamp, Inas H. Faris, Asma Boumaza, Roland Zerelles, and Giacomo Annio conducted MRI experiments and collected data. Valéria Bisio, Nicolas Dulphy, Karl Balabanian, and Andrea Federici provided and prepared spheroids. Gabrielle Mangin and Hannah Fels‐Palesandro performed data analyses and designed all figures. Valérie Vilgrain and Valérie Paradis provided assistance regarding data analysis and sample handling. Gabrielle Mangin, Hannah Fels‐Palesandro, Katharina Schregel, and Ralph Sinkus drafted the article. All authors revised the manuscript critically and approved its submission.

## Funding

This work was supported by funding from the integrated cancer research center “SiRIC InsiTu: Insights into cancer: From inflammation to tumor” (Grant Number INCa‐DGOS‐INSERM‐ITMO Cancer_18008). This work, performed within the context of the RHU Operandi, was supported in part by the French National Research Agency (Agence Nationale de la Recherche [ANR]) as its third PIA, integrated to France 2030 plan under reference (ANR‐21‐RHUS‐0012) (to Valérie Vilgrain). The study received funding from the Cancer Research UK (CRUK) Early Detection and Diagnosis (EDDCPGM\100001) Grant. This work was performed in collaboration with FRIM facility (UMS34), University Paris Cité, member of France Life Imaging (Grant ANR 11‐INBS‐0006) and IDEX Imagerie Du Vivant networks, and in collaboration with IAME group (UMR1137). Studies regarding the spheroids are supported by a grant from the ITMO Cancer of Aviesan within the framework of the 2021–2030 Cancer Control Strategy on funds administered by Inserm with Reference ASC23010HSA. This research was partially funded by the ITMO Cancer Aviesan/Inserm/Cancer 2020 Grant Ref. deSP/PB No. 241. Katharina Schregel received funding from the Else Kröner‐Fresenius Foundation (Grant Number 2023_EKEA.53). Hannah Fels‐Palesandro was supported by the Clinician Scientist Program of the Faculty of Medicine, Heidelberg University. Giacomo Annio was supported by the European Union's Horizon TMA MSCA Postdoctoral Fellowship Award (101068340).

## Ethics Statement

Experiments involving human‐derived materials (spheroids) were in accordance with the ethical standards of the responsible committee on human experimentation (institutional and national) and with the Helsinki Declaration of 1975, as revised in 2024. Ethics approval was obtained from the Ethics Review Committee for Biomedical Research Projects Paris Nord (CEERB, IRB # 00006477). Informed consent was obtained from all individuals. Experiments involving animals were approved by the French governmental authorities (Animal Protocol # 51789‐2024101411059997) and performed in compliance with the laboratory animal research guidelines/ARRIVE guidelines.

## Conflicts of Interest

The authors declare no conflicts of interest.

## Supporting information


**Video S1:** Illustration of the device with focus on the motor unit. When operating orthogonally in the main magnetic field B0 and applying a sinusoidal current, the dual‐coil motor does not rotate around its own axis but creates a translatory motion of the push–pull rod.
**Video S2:** Illustration of the transducer unit. The translatory back‐and‐forth motion of the push–pull rod is translated into a rotative motion of the transducer leading to an up–down motion.

## Data Availability

The data that support the findings of this study are available from the corresponding author upon reasonable request.
